# Is Carboxypeptidase B1 a Prognostic Marker for Ductal Carcinoma In Situ?

**DOI:** 10.3390/cancers13071726

**Published:** 2021-04-06

**Authors:** Charu Kothari, Alisson Clemenceau, Geneviève Ouellette, Kaoutar Ennour-Idrissi, Annick Michaud, Caroline Diorio, Francine Durocher

**Affiliations:** 1 Département de Médecine Moléculaire, Faculté de Médecine, Université Laval, Québec City, QC G1T 1C2, Canada; charu.kothari.1@ulaval.ca (C.K.); alisson.clemenceau.1@ulaval.ca (A.C.); genevieve.ouellette@crchudequebec.ulaval.ca (G.O.); 2 Centre de Recherche sur le Cancer, Centre de Recherche du CHU de Québec, Université Laval, Québec City, QC G1V 4G2, Canada; kaoutar.ennour-idrissi.1@ulaval.ca (K.E.-I.); Annick.Michaud@crchudequebec.ulaval.ca (A.M.); caroline.diorio@crchudequebec.ulaval.ca (C.D.); 3 Département de Médecine Sociale et Préventive, Faculté de Médecine, Université Laval, Québec City, QC G1T 1C2, Canada; 4 Centre des Maladies du Sein, Hôpital du Saint-Sacrement, Québec City, QC G1S 4L8, Canada

**Keywords:** breast cancer, DCIS, prognostic marker, ADH, IDC, human transcriptome array, early detection

## Abstract

**Simple Summary:**

Ductal carcinoma in situ (DCIS) is an early-stage breast cancer (BC), in which tumor cells are growing in a localized duct of the mammary gland. DCIS is considered a precursor disease for invasive BC and, therefore, treated as soon as it is identified. However, low-grade DCIS can be confused with atypical ductal hyperplasia, which is not a malignant lesion, leading to unnecessary surgery in around 70% of women with suspected DCIS. On the other hand, if left untreated, a DCIS has the potential to progress to IDC. In this retrospective study, we identified a gene signature, *carboxypeptidase B1* (*CPB1)*, the expression of which could help differentiate DCIS from an ADH lesion and DCIS that may progress to an invasive BC.

**Abstract:**

Ductal carcinoma in situ (DCIS) is considered a non-obligatory precursor for invasive ductal carcinoma (IDC). Around 70% of women with atypical ductal hyperplasia (ADH) undergo unnecessary surgery due to the difficulty in differentiating ADH from low-grade DCIS. If untreated, 14–60% of DCIS progress to IDC, highlighting the importance of identifying a DCIS gene signature. Human transcriptome data of breast tissue samples representing each step of BC progression were analyzed and high expression of *carboxypeptidase B1* (*CPB1*) expression strongly correlated with DCIS. This was confirmed by quantitative PCR in breast tissue samples and cell lines model. High *CPB1* expression correlated with better survival outcome, and mRNA level was highest in DCIS than DCIS adjacent to IDC and IDC. Moreover, loss of CPB1 in a DCIS cell line led to invasive properties associated with activation of *HIF1α*, FN1, STAT3 and *SPP1* and downregulation of SFRP1 and *OS9*. The expression of CPB1 could predict 90.1% of DCIS in a cohort consisting of DCIS and IDC. We identified CPB1, a biomarker that helps differentiate DCIS from ADH or IDC and in predicting if a DCIS is likely to progress to IDC, thereby helping clinicians in their decisions.

## 1. Introduction

Breast cancer (BC) progression is thought to progress in three different steps from normal breast cells to atypical ductal hyperplasia (ADH), ductal carcinoma in situ (DCIS), and invasive ductal carcinoma (IDC). However, the route of progression up to an IDC is controversial. There are several theories regarding BC progression: (i) ADH does not need to go through the DCIS step to progress to IDC; (ii) DCIS is not the precursor of IDC; the two diseases have different origins, and (iii) each DCIS has a genetic makeup/alteration required to become IDC and hence is considered a precursor for IDC [1]. Studies analyzing gene and protein expression patterns in all these different BC progression lesions have identified that the expression pattern of DCIS is very different from that of normal breast cells or ADH and the gene expression pattern of DCIS is altogether more similar to IDC [2,3,4]. Apart from the overlapping expression pattern from these studies, a previous study from our group also has identified a unique set of genes only expressed in DCIS or IDC, highlighting their difference from each other as well as from normal cells and ADH lesions [5].

DCIS is a stage of BC progression where abnormal cells (cancerous) appear in mammary ducts. DCIS is an early and pre-invasive BC stage where BC cells have not invaded the basement membrane of ducts and are still localized (in situ) [6]. DCIS presents different histopathological, clinical, and genetic changes than normal ducts. These changes increase significantly with the increase of the tumor grade of DCIS. As stated above, DCIS is mostly considered as a precursor of invasive BC, typically starting from ADH, and the changes seen in DCIS are represented in IDC [7].

It has been reported that around 30% of ADH upgrades to DCIS or IDC at the time of surgery, and similar data have been shown for a DCIS upgrading to IDC at the time of surgery [8,9,10]. This happens due to random sampling of radiological images leading to the identification of only ADH or DCIS and missing the accompanying lesions (DCIS and/or IDC, respectively). Moreover, studies have shown that women with an ADH lesion have a 3–5-fold higher risk of developing BC [11], and around 14–60% of DCIS are likely to progress to IDC if left untreated [7]. This highlights the crucial need to properly differentiate an ADH lesion from a low-grade DCIS, as this could result in unnecessary surgical interventions in 70% of cases [5].

In the present study, we identified *carboxypeptidase B1* (*CPB1*) as a gene highly expressed in DCIS than ADH, DCIS adjacent to IDC, and IDC. This gene could serve as a biomarker to differentiate a DCIS from an ADH lesion, as well as identify a DCIS with the potential to progress to IDC. This has important clinical implications to aid clinicians in their decisions regarding women presenting with an ADH or a DCIS lesion and which women could be spared from surgery and benefit from a close follow-up.

## 2. Materials and Methods

### 2.1. Breast Tissue Samples Selection

Breast lesion tissue samples were provided from the tissue bank located at the Center des Maladies du Sein of the Hôpital du Saint-Sacrement, Quebec, Canada. For this study, we selected patients (N = 82) with breast tissue samples deposited in the biobank from 2010 to 2014 with no hormonotherapy or chemotherapy treatment before surgery. Clinical data, including age at mastectomy and menarche, menopausal status, body mass index (BMI), and presence of microcalcifications on surgical specimen often associated with DCIS, were collected from medical reports. All breast diseases were confirmed by an experienced breast pathologist, and all tumor characteristics were routinely collected from medical reports: tumor size, histologic type, grade, lymph node involvement, and receptor status (ER: estrogen receptor, PR: progesterone receptor, and HER2: human epidermal growth factor receptor 2). Formalin-fixed paraffin-embedded (FFPE) blocks containing DCIS (N = 13) and IDC (N = 69) were selected by a pathologist specialized in breast pathology.

### 2.2. Tissue Microarray (TMA) Preparation

TMAs were constructed from the FFPE mastectomy blocks containing lesional/tumor tissue samples. BC was identified by a pathologist. Molecular classification of tumors was based on the ER, PR, and HER2 immunohistochemistry (IHC) analysis of the slides prepared from mastectomy blocks. For each tissue sample, 2 cores of 1 mm diameter were randomly arrayed on a virgin paraffin TMA block using a tissue microarrayer (Estigen tissue science, Estigen OÜ, Tiigi 61b, 50,410 Tartu, Estonia). Slides were prepared from the final TMA (4 µm thick section). The presence of cancerous tissue was confirmed on hematoxylin–eosin (H&E) stained slides.

### 2.3. Cell Lines

The MCF10A cell line series was developed to represent the different steps of BC progression [12,13]; MCF10A (normal/benign), MCF10AT1 (ADH), MCF10DCIS.com (DCIS) and MCF10CA1a (IDC). This series was used to validate the findings obtained from breast tissue samples analysis and to understand the role of CPB1 in DCIS in vitro. The HEK293T cell line was used for transfection and immunoprecipitation experiments. DMEM/F12 Ham’s mixture (1:1) medium was used for MCF10A cell line series supplemented with 1% HEPES, 5% horse serum. MCF10A and MCF10AT1 medium were additionally supplemented with 0.002 mg/mL insulin, 0.5 µg/mL hydrocortisone, and 0.02 µg/mL epidermal growth factor. The HEK293T cell line was grown in DMEM media supplemented with 10% fetal bovine serum. All media were supplemented with 1% penicillin–streptomycin mixture (5000 IU penicillin, 5000 μg/mL). All cell culture reagents were purchased from Wisent Inc. (Québec, QC, Canada).

### 2.4. RNA Extraction and Quantitative Real-Time PCR (qPCR) Analysis

Total RNA from breast tissue samples and MCF10A cell lines was isolated with Qiagen RNeasy FFPE Kit and Qiagen RNeasy mini kit (Qiagen, Hilden, Germany), respectively. Preparation of RNA samples for whole-genome expression analysis was performed using the SensationPlus™ FFPE amplification kit (Affymetrix, Thermo Fisher Scientific Waltham, MA, USA).

### 2.5. Quantitative PCR Was Performed Using SyBr Green Technology as Described Previously

Briefly, oligo-primer pairs that allow the amplification of ~100 to 150 base pairs (bp) of the indicated specific mRNA (*CPB1*, *SFRP1*, *OS9*, *HIF1α*, *FN1*, *SPP1*, *ATP50*, *GAPDH*, and *HPRT1*) were designed with the GeneTools 2.0 software (Biotools Inc., Edmonton, AB, Canada), and their specificity was verified by blasting the GenBank database [14]. The sequence of all primers is indicated in Table S1. Data calculation and normalization were performed using the second-derivative and double-correction method [15], with housekeeping genes (*ATP50*, *HPRT1*, and *GAPDH*). The mRNA levels were expressed as the number of copies/µg of total RNA calculated using corresponding standard curves.

### 2.6. Analysis of Public BC Datasets

The difference in *CPB1* expression pattern between the normal breast tissue and tumoral tissue samples as well as along different molecular subtypes of BC (luminal A, luminal B, HER2+, and triple-negative BC (TNBC)) was analyzed using data from gene expression databases of normal and tumor tissues (http://gent2.appex.kr/gent2/, accessed on 5 December 2020). The significance analysis for the comparison of *CPB1* expression was done with GraphPad Prism 8. For normal breast vs. BC tissue samples, an unpaired Student’s t-test was used, whereas Kruskal–Wallis one-way analysis of variance was used to determine the significance of the differences observed in the expression pattern of *CPB1* in different molecular subtypes. The term basal is used for the set of tissue samples with basal characteristics but could not be classified as TNBC.

Furthermore, the effect of *CPB1* expression on survival was analyzed using Kaplan–Meier plotter (https://kmplot.com/analysis/, accessed on 20 November 2020). Relapse free survival (RFS; N = 3951), overall survival (OS; N = 1402), distance metastasis-free survival (DMFS; N = 1803) and post-progression survival (PPS; N = 414) were analyzed.

### 2.7. Lentiviral Production and Infection

Five lentiviruses (TRCN0000046873, TRCN0000046874, TRCN0000046875, TRCN0000046876, and TRCN0000046877) were tested against CPB1, out of which two (TRCN0000046874 and TRCN0000046876) were used for further analysis. The two shRNA used for the analysis are labeled as shCPB1-874 (TRCN0000046874) and shCPB1-876 (TRCN0000046876) throughout the manuscript. The shRNAs were a kind gift from Prof. S. Gobeil, Université Laval, Québec, Canada. Lentivirus packaging was done in HEK293T cells plated in 60–70% confluency in 10 cm tissue culture plates. The shRNA and shScrambled in pLKO.1-puro vectors were co-transfected with pMD2.G (VSVG coding plasmid) and psPAX2 (lentivirus packaging plasmid) using Polyplus-transfection^®^ jetPRIME^®^ (Polyplus-transfection S.A, Illkirch, France). After 12–16 h of transfection, the media was changed. The media with the virus was collected at 24 h and 48 h. The virus-containing media was filtered using a 0.45 µm pore size filter (MilliporeSigma, Darmstadt, Germany). The virus was aliquoted and stored at −80 °C until further use. For infection, the cells were infected with the virus in culture media containing 10 µg/mL polybrene. The media was changed after 16 h of incubation at 37 °C at 5% CO_2_. After 24 h of further incubation, the cells were split with 1 µg/mL of puromycin. Cells infected with shScrambled are labeled as shControl throughout the manuscript.

### 2.8. Proliferation

The MCF10DCIS.com control cell line (shControl) and with CPB1 knockdown (KD) were seeded in 96-well plates (10,000 cells/well). After 72 h of incubation at 37 °C with 5% CO_2_, 100 µL of culture media containing 10% alamarBlue^®^ (Thermo Fisher Scientific, QC, Canada) was added, and the plates were incubated at 37 °C with 5% CO_2_ until the first appearance of pink color. The absorbance was measured at 570 nm and 600 nm wavelengths. The experiments were performed in triplicates and repeated three times.

### 2.9. Migration

MCF10DCIS.com shControl and CPB1 KD cells were seeded in 2 chamber wells (Ibidi GMBH, Martinsried, Planegg, Germany) and incubated at 37 °C with 5% CO_2_. After 24 h, the chamber was removed, and the culture media was replaced with a culture media containing 10 µM mitomycin C. Images were taken at 0 h, and migration was followed until the gap between the growing cells was filled. The experiments were repeated three times. The images were taken with the EVOS™ M5000 imaging system (Invitrogen™, Thermo Fisher Scientific, Waltham, MA, USA).

### 2.10. Spheroid Assay

MCF10DCIS.com CPB1 KD and shcontrol cells were seeded in Corning^®^ 96-well clear round-bottomed ultra-low attachment microplates (5000 cells/well, Millipore Sigma, Oakville, ON, Canada). The cells were incubated at 37 °C with 5% CO_2_ for 7 days. The experiments were performed in triplicates and were repeated three times. The images were taken with the EVOS™ M5000 imaging system (Invitrogen™, Thermo Fisher Scientific, Waltham, MA, USA).

### 2.11. Flag Immunoprecipitation (IP)

Transfection: HEK239T cells were transfected with either 8 µg of pcDNA3.1-CPB1-flag or pcDNA3.1–3*flag. The media was changed after 6 h of transfection. After 24 h of incubation at 37 °C with 5% CO_2_, the cells were lysed with lysis buffer (50 mM HEPES, 150 mM NaCl, 10% glycerol, 1.5 mM MgCl_2_, 0.1% NP-40, 1 mM DTT, 1 mM PMSF, 200 mM Na_3_VO_4_ and 1× protease inhibitor cocktail). The lysate was vortexed and incubated on ice for 10 min with intermittent vortexing. After 10 min, the lysate was centrifuged for 10 min at 13,000 rpm at 4 °C. The supernatant was collected, and protein estimation was done by Bradford assay. For immunoprecipitation, 1 mg/mL of protein was utilized. From each sample, 10 µg protein was aliquoted in a fresh tube as input for Western blotting.

Protein A/G bead preparation for pre-clearing and final IP: For each reaction, 20 µL and 60 µL of the slurry containing 1:1 concentration of beads were taken pre-clearing and final IP, respectively. The slurry was centrifuged for 1 min at 5000 rpm at 4 °C. The beads were washed 3 times with 250 µL of lysis buffer as described above.

Pre-clearing of the protein lysate: beads prepared for pre-cleaning were added to the protein lysate and were incubated at 4 °C for 2 h in a rotator. After incubation, the mixture was centrifuged at 10,000 rpm for 1 min at 4 °C. The supernatant was collected for IP.

IP: the supernatant from pre-clearing was incubated with 15 µL of Flag antibody and was incubated at 4 °C for 2 h on the rotator. After the incubation, the beads prepared for the final IP were added to the samples and were incubated at 4 °C overnight in a rotator. After incubation, the samples were centrifuged at 5000 rpm for 1 min at 4 °C. The supernatant was discarded, and the beads were washed 5 times with 250 µL of lysis buffer. A portion of IP beads was aliquoted for Western blot analysis for confirmation of IP.

### 2.12. Western Blotting

Loading buffer was added to the protein samples (input/other protein samples = 1 × final concentration; aliquoted IP beads = 25 µL of 1× loading buffer/sample) and was incubated at 95 °C for 5 min. The samples were briefly centrifuged and analyzed on SDS-polyacrylamide gel. Western blotting was done with a standard protocol using nitrocellulose membrane. Blocking was performed in 5% milk (no-fat) in TBS-Tween.

### 2.13. Mass Spectrometry

The beads aliquoted for mass spectrometry were washed several times with 1× PBS-containing protease inhibitors (1 mM DTT, 1 mM PMSF, 200 mM Na_3_VO_4_, and 1× protease inhibitor cocktail). After washing, the beads were washed 5 times with 50 mM ammonium bicarbonate (pH 8). Excess buffer was removed, and beads were sent to the proteomic platform of Centre de recherche du CHU de Québec-Université Laval, Canada, for mass spectrometry analysis.

### 2.14. IHC Analysis

Deparaffinization of slides was done using toluene followed by rehydration with decreasing concentrations of ethanol. Antigen retrieval was done in preheated TEG buffer at 95.6 °C in a water bath for 30 min, followed by 15–20 min incubation at room temperature (RT) to gradually decrease the buffer temperature. After washing 3 times, endogenous peroxidase and nonspecific binding were blocked using a 3% H_2_O_2_ bath for 15 min. Slides were washed (3 times) and were blocked using Superblock (IDetect™ super stain system HRP, London, ON, Canada) for 1 h at RT. Slides were washed (3 times) and were incubated with the primary antibody of CPB1 (anti-rabbit, cat no. 12600-1-AP, Proteintech, Rosemont, IL, USA) at 1:600 dilution in DAKO antibody diluent and incubated overnight at 4 °C inside a moisture chamber. Slides were then incubated with the secondary antibody (Dako, EnVision™ + dual-link system-HRP, Santa Clara, CA, USA) at RT for 30 min, followed by 3 washes. The staining was done by incubating the slides for 2 min at RT with chromogen substrate 3,3′-diaminobenzidine (DAB; Empire Genomics, Buffalo, NY, USA). The slides were washed with distilled H_2_O (3 times) and were counterstained using Harris’s hematoxylin (Intermedico, Markham, ON, Canada), followed by 5 washes with running H_2_O and dehydration using ethanol and xylene. The slides were then mounted and allowed to dry before scanning. All washes were done using 1 × Tris-buffered saline for 5 min except otherwise indicated.

The assessment of CPB1 staining was done by analyzing the percent of cancer cells stained, the intensity of staining, and the heterogeneity of staining intensities. Scoring for intensity corresponds to 0 = no staining; 1 = low-intensity; 2 = medium-intensity; 3 = high-intensity. The H-score was calculated as follows: (percent of cells stained with low-intensity × 1) + (percent of cells stained with medium-intensity × 2) + (percent of cells stained with high-intensity × 3). Examples of scoring are presented in Figure S1. Reproducibility of the scoring was examined by independent scoring of 10% randomly selected TMAs by a pathologist (r > 0.6). The median H-score for CPB1 expression was used as a cutoff for the statistical analysis.

### 2.15. Statistical Analysis

All IHC data analyses were performed with RStudio v1.2.5033 (RStudio Team (2019), RStudio: Integrated Development for R, RStudio, Inc., Boston, MA, USA, http://www.rstudio.com/). Comparisons of means between two groups were performed using a *t*-test for independent samples, with the *t*-test function of the R package stats v3.6.2. Comparisons of proportions between groups were performed using a khi2 test with the chisq.test function of the R package stats v3.5.1. Boxplots were drawn with the boxplot function of the R package graphics v3.6.2. Hypothesis testing, both univariate and multivariate, were performed by a generalized linear model of regression with the glm function of the R package stats v3.6.2. Logistic regression was used to predict binary outcome (DCIS versus IDC) from a set of continuous predictive variables (CPB1 expression, age at mastectomy, and BMI). The receiver operating characteristic (ROC) curves were drawn, and the area under the curve (AUC) was obtained with the plot.roc and roc functions of the R package pROC v1.16.1, respectively. *P*-values lower than 0.05 were considered significant.

## 3. Results

### 3.1. HTA Analysis Identified CPB1 as Specifically Expressed in DCIS

**Figure 1:**
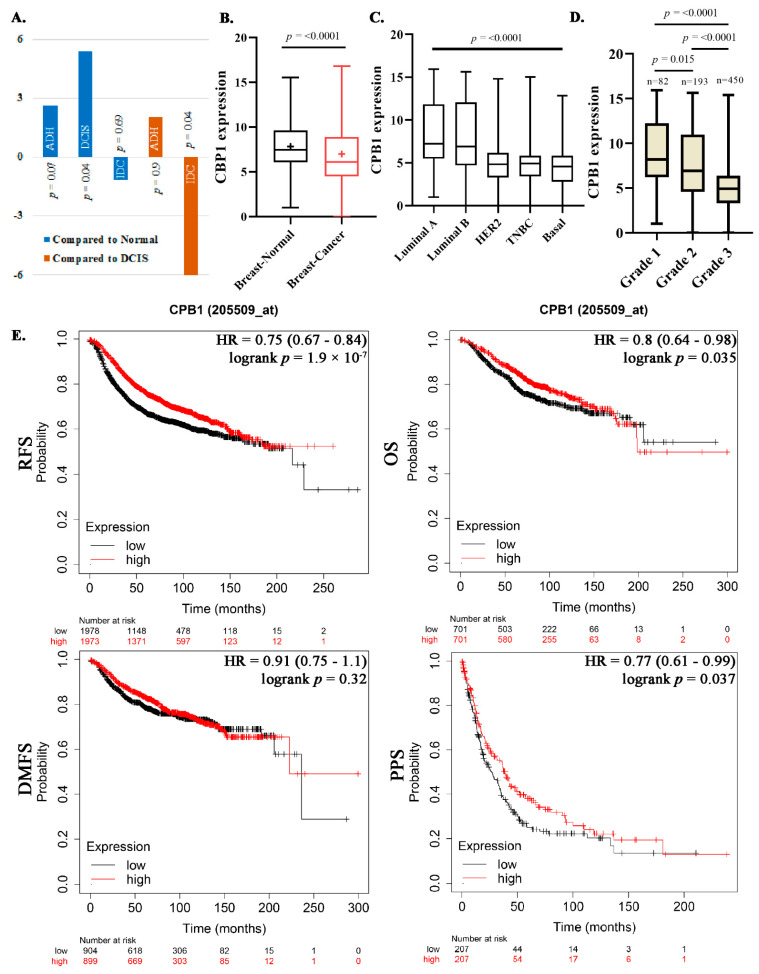
Expression of carboxypeptidase B1 (CPB1) in human transcriptome array (HTA) data and breast cancer (BC) datasets. Fold change in the expression level of *CPB1* gene according to HTA analysis. Five samples for each step of BC progression were used for HTA analysis. *p*-value was determined by one-way between-subject ANOVA (**A**). The expression level of *CPB1* in breast cancer tissue samples (N = 5574) than normal breast tissue samples (N = 474). *p*-value was calculated by unpaired Student’s *t*-test (**B**). The expression level of *CPB1* across different BC molecular subtypes of BC patients: luminal A (N = 379), luminal B (N = 244), HER2 (N = 230), TNBC (N = 251) and basal (N = 363) in a gene expression database (GENT2) dataset. The *p*-value was calculated using Kruskal–Wallis one-way analysis of variance (**C**). The difference in *CPB1* expression pattern according to the tumor’s grade in the GENT2 dataset (**D**). Effect of *CPB1* on survival outcome of BC patients: RFS = reversal free survival; OS = overall survival; DMFS = distance metastasis-free survival; PPS = post-progression survival (**E**).

To identify a gene signature that could differentiate DCIS from ADH and IDC, we reanalyzed HTA data from our previous study [5] and observed the highest expression of *CPB1* in DCIS than other lesions. The HTA data highlighted that the expression of *CPB1* was 5.40-fold (*p*-value = 0.04) higher in DCIS than normal, whereas we also found a 2.62-fold increase in ADH, but this was not a statistically significant difference (*p*-value of 0.07; Figure 1A). As for IDC, it showed a decrease in *CPB1* expression; −1.2-fold (*p*-value = 0.69) than normal tissue, and −6.44-fold (*p*-value = 0.04) than DCIS (Figure 1A).

### 3.2. Association of CPB1 with Better Survival Outcomes

We then compared the expression pattern of *CPB1* in normal breast tissues (N = 475) vs. BC tissues (N = 5574) from GENT2 dataset and found a slight decrease in overall {mean: 7.8 (normal), 7.02 (cancer); median: 7.47 (normal), 6.13 (cancer)} expression of *CPB1* in BC patients (*p*-value < 0.0001) (Figure 1B). To understand the differential expression of *CPB1* in BC patients, we stratified the BC cohort according to BC subtypes: luminal A (N = 379), luminal B (N = 244), HER2 (N = 230), TNBC (N = 251) and basal (N = 363). The results of this stratification suggest that the expression of *CPB1* may be higher in low-grade tumors (luminal A and luminal B) than in other subgroups (*p*-value < 0.0001) (Figure 1C). To confirm this, we stratified the BC dataset from a gene expression database (GENT2) consisting of 725 BC samples according to grade and observed a mean expression of *CPB1* of 8.84, 7.54, and 5.55 in grade 1, 2, and 3, respectively (*p*-value < 0.0001; Figure 1D).

The high expression of *CPB1* in low-grade tumors prompted us to look at the survival outcome for patients expressing *CPB1*. The survival analysis shows that *CPB1* expression correlated with better survival outcomes for relapse-free survival (RFS) (*p*-value = 1.9 × 10^−7^), overall survival (OS) (*p*-value = 0.035), and post-progression survival (PPS) (*p*-value = 0.037) with the exception of distant metastasis-free survival (DMFS) where the data were not statistically significant (*p*-value = 0.32) (Figure 1E).

### 3.3. Validation of CPB1 Expression in DCIS

**Figure 2:**
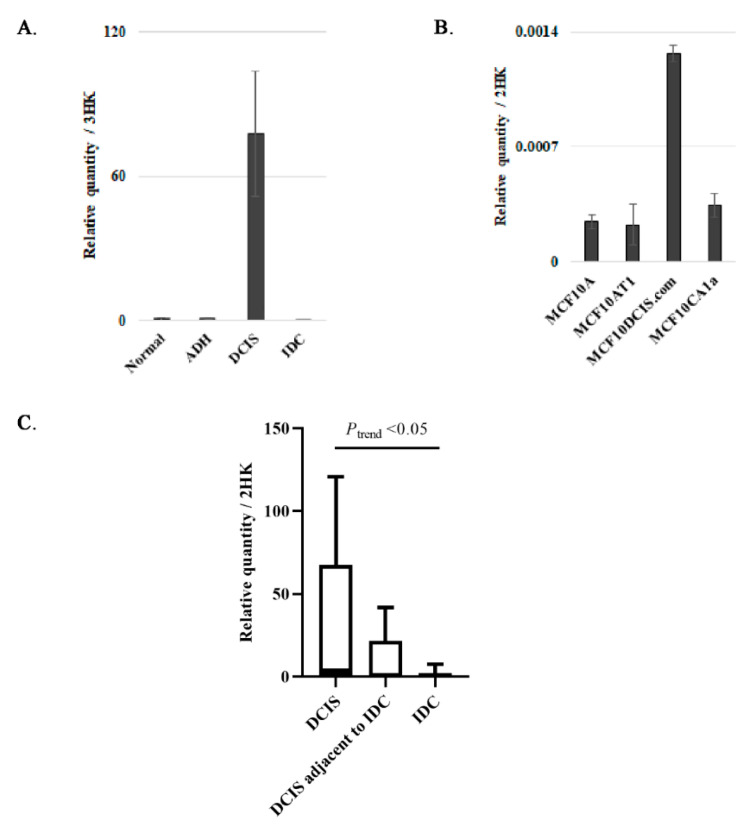
qPCR Validation of *CPB1* expression in breast samples and cell lines. *CPB1* mRNA expression in different BC progression steps (normal, ADH, DCIS, and IDC) (**A**). *CPB1* expression pattern in MCF10A cell line series: MCF10A (normal); MCF10AT1 (ADH); MCF10DCIS.com (DCIS) and MCF10CA1a (IDC). The graph is representative of 3 independent experiments (**B**). *CPB1* expression in breast tissue samples: DCIS (5 samples), DCIS adjacent to IDC (5 samples), and IDC (10 samples). The qPCR data were log transformation to obtain a “normal distribution”, and a linear test between histological types and log(qPCR) was performed to obtain the *p*-value for the trend. In each case where a lesion is mentioned adjacent to another lesion, the first was taken for analysis. The term “adjacent to” refers to a second lesion also present in the surgical specimen (**C**). All expression analyses were performed by quantitative real-time PCR (qPCR). *CPB1* expression was normalized to 2 (*HPRT1* and *GAPDH*) or 3 (*ATP50*, *HPRT1*, and *GAPDH*) housekeeping genes. ADH = atypical ductal hyperplasia; DCIS = ductal carcinoma in situ; IDC = invasive ductal carcinoma.

We then went on to answer the question as to whether the expression of *CPB1* changes as the tumor progresses. To answer this question, we first performed qPCR analysis for expression of *CPB1* in different steps of BC progression. We performed qPCR on 3 breast tissue samples used in HTA analysis for normal, ADH, DCIS, and IDC samples. We confirmed that *CPB1* expression was significantly higher in DCIS than in other samples (Figure 2A). We also obtained similar results in the MCF10A cell line model of BC progression (Figure 2B).

Next, we performed qPCR for *CPB1* in breast tissue samples different from those used in HTA or IHC analysis: DCIS (*n* = 5) from women diagnosed with no other breast disease (DCIS), DCIS (*n* = 5) from women diagnosed with an IDC (DCIS adjacent to IDC), and IDC (*n* = 10) from 5 women also diagnosed with DCIS and 5 women with no other breast disease (IDC) (Figure 2C). All the IDC samples were grouped for analysis because the expression was similar. We found that *CPB1* expression was highest in DCIS, when DCIS was the worst diagnosed lesion, followed by DCIS adjacent to IDC and barely expressed in IDC (*P*_trend_ < 0.05). These data suggest that *CPB1* expression is specific to DCIS that has not led to IDC.

### 3.4. Knockdown of CPB1 Leads to an Increase in the Invasive Properties of the DCIS Cell Line

**Figure 3:**
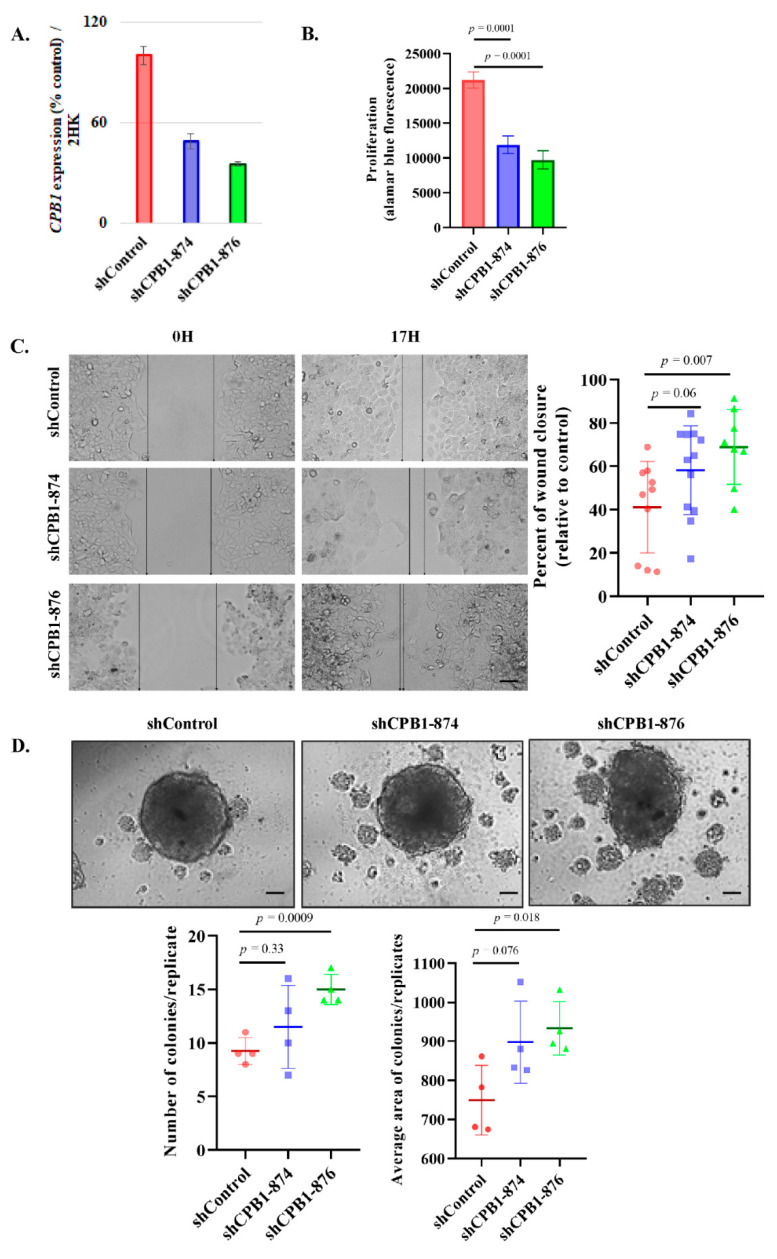
Effect of *CPB1* on Ductal carcinoma in situ (DCIS) phenotype. Effect of *CPB1* knockdown on *CPB1* mRNA expression (**A**), proliferation (**B**), migration (**C**), and 3D colony formation by spheroid assay (**D**). Two different shRNAs against *CPB1* were used for all analyses. Images are representative of three independent biological replicates. The *p*-values were calculated by unpaired Student’s *t*-test. All assays were performed in the MCF10ADCIS.com (DCIS) cell line. Scale bar 100 µm.

To understand the role of CPB1, we knocked down *CPB1* expression using two different shRNAs, achieving around 51% and 65% reduction in *CPB1* expression (shCPB1-874 and shCPB1-876, respectively; Figure 3A) in the MCF10ADCIS.com cell line. We further analyzed the effect of *CPB1* knockdown on proliferation, migration, and 3D colony formation (spheroid assay) in MCF10ADCIS.com cell line. Results show 43.8% (*p*-value = 0.0001) and 54.1% (*p*-value = 0.0001) decrease in proliferation (Figure 3B), and 16.3% (*p*-value = 0.06) and 27.08% (*p*-value = 0.007) increase in migration with shCPB1-874 and shCPB1-876, respectively than control (Figure 3C). Furthermore, we found an increase in both the number and average area of colonies formed in 3D colony formation. KD with shCPB1-874 increased the number of colonies by 24.3% (*p*-value = 0.33) and the average area of colonies by 19.8% (*p*-value = 0.076). As for shCPB1-876, an increase of 62.16% (*p*-value = 0.0009) in the number of colonies and 24.5% (*p*-value = 0.018) in average size of colonies was observed (Figure 3D).

These results show that a decrease in *CPB1* expression in the MCF10DCIS.com cell line alters their phenotype to become more migratory and tumorigenic. On the other hand, there is a decrease in the proliferation rate.

### 3.5. Changes in Signaling after Knockdown of CPB1 in MCF10DCIS.com Cell Line

**Figure 4:**
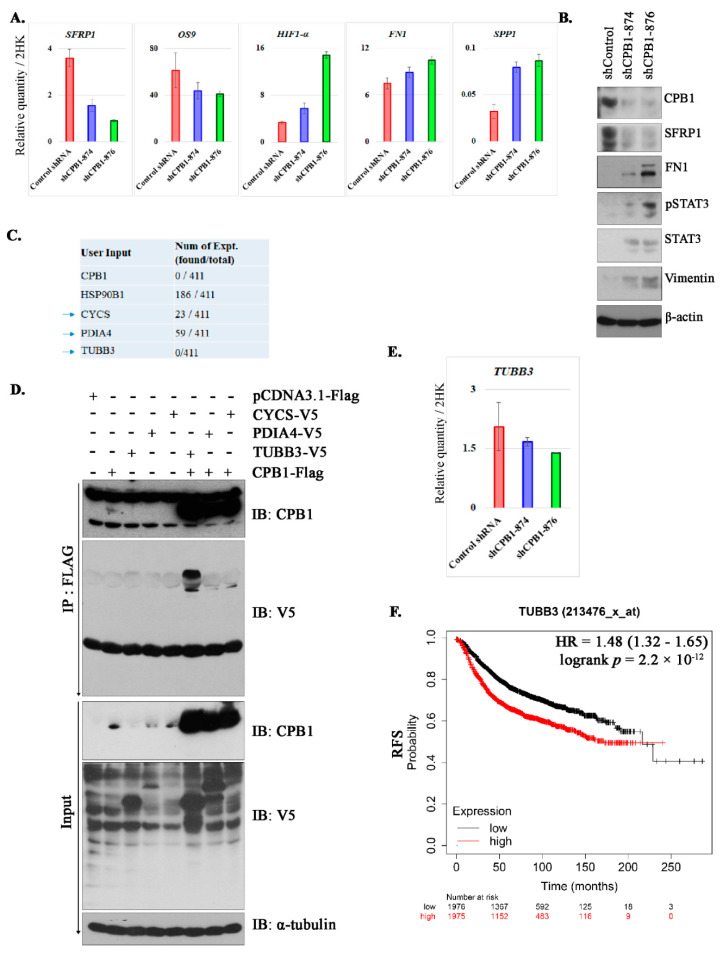
Signaling altered by CPB1 knockdown in MCF10ADCIS.com cell line and its interactome. Effect of CPB1 knockdown on mRNA expression of *SFRP1* (tumor suppressor gene), *OS9* (inhibitor of HIF1α) and genes with oncogenic potential (*HIF1α*, *FN1*, and *SPP1*) (**A**), the protein level of SFRP1, FN1, phosphor/total STAT3, and vimentin (**B**) of signaling molecules. CRAPome analysis of interactors of CPB1 identified by AP-MS analysis in HEK293T cell line, arrows indicate the proteins selected for validation, namely: CYCS: cytochrome C somatic, PDIA4: protein disulfide isomerase family A member 4, and TUBB3: tubulin beta 3 class III (**C**). Validation of true interactors of CPB1 by co-immunoprecipitation in HEK293T cells, indicating TUBB3 as a direct interactor of CPB1 (**D**). Effect of CPB1 knockdown on mRNA level of *TUBB3* (**E**). Effect of *TUBB3* on RFS of BC patients; HR = hazard ratio (**F**). All figures are representative images of two biological repeats.

The pronounced effect of the depletion of CPB1 on the migration and 3D colony-formation prompted us to investigate its underlying mechanism. We analyzed the effect of CPB1 both as a tumor suppressor and in oncogenic signaling. Interestingly, results show decreased tumor suppressor gene secreted frizzled related protein 1 (SFRP1) upon depletion of CPB1 expression at both mRNA and protein levels (Figure 4A,B). We also found a significant increase in *HIF1α* (hypoxia-inducible factor 1-alpha) expression with a decrease in the inhibitor of HIF1α, *OS9* (Osteosarcoma Amplified 9, Endoplasmic Reticulum Lectin) (Figure 4A). Furthermore, we also observed a pronounced increase in FN1 (fibronectin 1; both mRNA and protein), *SPP1* (secreted phosphoprotein 1), STAT3 (signal transducer and activator of transcription 3), and vimentin (Figure 4A,B). These results suggest that CPB1 KD downregulates the tumor suppressor signal (SFRP1) and upregulates the signaling molecules related to increasing migration and tumorigenesis (FN1, *SPP1*, STAT3, vimentin, and *HIF1α*).

We further investigated the interactors of CPB1 by affinity-purified mass spectrometry. Due to the low copy of CPB1 in the mass-spectrometric analysis, only 4 interactors were identified. We analyzed these interactors in the Contaminant repository for affinity purification (CRAPome) database to identify the interactors normally pulled down with flag-tag (Figure 4C). Based on the results from CRAPome analysis, we selected 3 proteins for validation: CYCS, PDIA4, and TUBB3. IP analysis identified TUBB3 as a true interactor of CPB1 (Figure 4D). We then pursued to understand what happens to *TUBB3* expression when we knockdown CPB1. The qPCR data show a decrease in the mRNA level of *TUBB3* upon CPB1 KD (Figure 4E). Furthermore, we analyzed the effect of *TUBB3* expression in BC patients from an online dataset (https://kmplot.com/, accessed on 6 December 2020) and found that *TUBB3* expression is associated with poor RFS in BC patients (Figure 4F).

### 3.6. Characterization of the Study Population Based on CPB1 Expression by IHC

**Figure 5:**
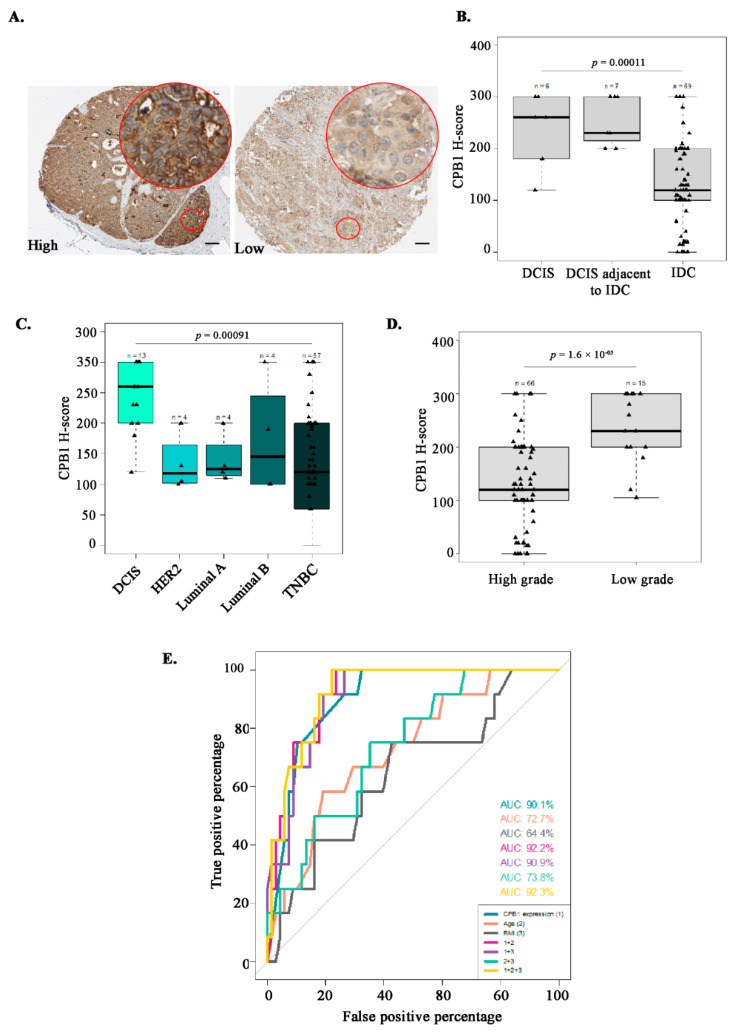
Analysis of CPB1 expression in BC cohorts. Representative images of CPB1 staining by immunohistochemistry (IHC) in breast tissue samples: high > 130, low < 130 (**A**). Scale bar 100 µm. Expression pattern of CPB1 in DCIS, DCIS adjacent to invasive ductal carcinoma (IDC) and IDC patient samples based on H-score (**B**), in different molecular subtypes of BC (**C**), and according to the grade of BC (**D**). Prediction model for breast cancer subtypes based on the expression pattern of CPB1 by receiver operating characteristics (ROC) curve schematization (**E**).

IHC staining of CPB1 was performed on the entire cohort (N = 82). We stratified our study population based on the median H-score of CPB1 expression: low CPB1 expression ≤ 130 (N = 43), and high CPB1 expression > 130 (N =39) (Table 1, Figure 5A). No significant difference was observed considering age at mastectomy (*p*-value = 0.22) and menarche (*p*-value = 0.08). However, we found that only 48.7% (19/39) were postmenopausal women in the high-CPB1 expressing group than 74.4% (34/43) postmenopausal women in the low-CPB1 expressing group (*p*-value = 0.03). As an increase in BMI is generally linked with postmenopausal women, we compared the expression of CPB1 with BMI and observed that the average BMI was lower in the high-CPB1 expression group (25.5 ± 5.30) than the low-CPB1 expression group, where mean BMI was 28.6± 5.7 (*p*-value = 0.01; Table 1). We then examined IHC data to see whether results were concordant with qPCR results regarding the difference in expression of CPB1 in DCIS, DCIS adjacent to IDC, and IDC (Figure 2C). Our data showed higher expression of CPB1 in DCIS than in IDC (6 DCIS, 7 DCIS adjacent to IDC, and 69 IDC samples), with a *p*-value of 0.00011 (Figure 5B). We further stratified IDC (N = 69) according to molecular subtypes (i.e., HER2, luminal A, luminal B, and TNBC) and found that the expression of CPB1 was still highest in DCIS (N = 13; *p*-value = 0.00091; Figure 5C). The clinicopathological information of patients with DCIS is described in Table S2.

In addition, our data also underline the fact that high CPB1 expression is associated with low-grade BC. Out of the 39 patients with high CPB1 expression, we observed that 2.6% (1/39) were of grade 1, 30.8% (12/39) were of grade 2, and 66.7% (26/39) were of grade 3 tumors. On the other hand, out of 42 low CPB1 expressing patients, 0% (0/42) were of grade 1, 4.8% (2/42) were of grade 2, and 95.2% (40/42) were of grade 3 tumors. This difference was statistically significant (*p*-value = 0.003; Table 1). This is also represented in Figure 5D, where low-grade BC represents grades 1 and 2, and high-grade BC represents grades 3 (*p*-value = 1.6 × 10^−5^). As grading for DCIS and IDC is different, we examined CPB1 expression in low vs. high-grade DCIS and IDC separately and found a similar pattern for DCIS only (*p*-value = 0.33) and IDC only (*p*-value = 0.06), although these associations were not significant (Figure S2A,B). A higher proportion of patients with microcalcifications was found in the high CPB1 expression group (33.3%) compared with the low CPB1 expression group (20.9%) but without a significant difference between both groups (*p*-value = 0.33). The CPB1 score of expression was higher in the presence of microcalcifications (N = 22) compared with in absence of microcalcification (N = 60; *p*-value = 0.051; Figure S3A).

### 3.7. Association Studies Confirm CPB1 as a Predictive Marker of DCIS

CPB1 expression in tumoral lesions of all women (N = 82) was associated with DCIS before (odds ratio (OR) = 1.02; 95% confidence interval (CI) = 1.01–1.03; *p*-value < 0.001, Table 2) and after adjustment for age at mastectomy (OR = 1.02; 95% CI = 1.01–1.03; *p*-value < 0.001), and for age at mastectomy and BMI (OR = 1.02; 95% CI = 1.01–1.04; *p*-value < 0.001). CPB1 expression was still significantly associated with DCIS after stratification for the menopausal status (Table 2) and the presence of microcalcifications (Figure S3B), both in univariate and multivariate analyses. To evaluate the ability of CPB1 expression to identify DCIS from IDC, we performed receiver operating characteristics (ROC) curves (Figure 5E). The area under the curve (AUC) indicated that CPB1 expression alone was able to identify 90.1% of the DCIS (N = 12) present in the study cohort (N = 82). After the inclusion of age at mastectomy and BMI, the model identified 92.3% of the DCIS. Interestingly, after stratification for the presence of microcalcifications (N = 21), CPB1 expression alone identified 93.3% of the DCIS (N = 8) in the group of patients having microcalcifications and 100% after the inclusion of age at mastectomy and BMI into the model (Figure S3C). On the other hand, CPB1 expression alone identified 88% of the DCIS (N = 4) in the group of patients without microcalcifications and 93% after adjustment for the age at mastectomy and the BMI (Figure S3D).

## 4. Discussion

DCIS itself is generally not considered a life-threatening disease, and approximately 3% of females with DCIS will die of BC 20 years on average after diagnosis, and the risk is higher in young and black women [16,17]. Studies examining autopsy samples suggest that in several patients, a DCIS reservoir is present in the benign tissues. This does not become apparent until death, and it is only diagnosed if multiple sampling slides are prepared [10,18,19,20,21]. These reservoirs lead to the reoccurrence of DCIS in women treated for DCIS even after 15 years of follow-up [22,23,24,25]. The difficulty in differentiating low-grade DCIS from ADH results in many instances of misinterpretation of ADH lesions as a DCIS [26], often leading to overdiagnosis resulting in unnecessary surgery in around 70% of women diagnosed with ADH lesions at biopsy [8]. On the contrary, missing the DCIS repository due to low sampling may predispose to invasiveness as DCIS is considered a non-obligatory precursor of IDC [17,27]. Studies highlight that 50% of cases of recurrence in patients treated for DCIS are invasive, and these invasive diseases show genetic, morphological, and IHC similarities with original DCIS [28,29]. Therefore, early identification of DCIS is extremely important for improving the OS of BC patients.

With the hypothesis that each step of BC progression must have a gene signature and intending to identify a signature specific to DCIS, we analyzed HTA data from our previous study [5]. We identified that the DCIS step displayed the highest expression of *CPB1* than other steps of BC progression. CPB1 is a carboxypeptidase, with a catalytic activity releasing C-terminal lysine or arginine amino acid of proteins (https://www.uniprot.org/uniprot/P15086, accessed on 9 December 2020). Its role has been studied in the activation of the complement pathway of innate immunity [30].

The present study suggests that *CPB1* expression decreases as the severity of BC increases (Figure 1C,D), and its expression correlates with better survival outcomes in BC patients (Figure 1E). This correlates with a study by Swaisgood et al. 2002, which shows that CPB1 inhibits plasminogen’s role in fibrinolysis and cell migration by removing the lysyl residue at the c-terminal end [31]. The role of plasminogen in BC aggressiveness and its association with poor survival outcome has been shown [32,33,34]. Although Bouchal et al. 2015 have shown that high expression of *CPB1* is associated with good RFS among women diagnosed with luminal B tumors, they have also found that high expression of *CPB1* is associated with poor RFS among women diagnosed with luminal A tumors [35]. Compared with the grade of disease, CPB1 RNA and protein expressions were both correlated with low-grade tumors (Figure 5D and Figure S2A,B). Present data confirm that CPB1 expression was highest in DCIS than DCIS adjacent to IDC or all subtypes of IDC (Figure 5B,C). However, our data also show a difference in the RNA and protein expression of CPB1, where we see that RNA expression of *CPB1* is only present in DCIS (Figure 2A,C). On the other hand, protein expression of CPB1, though highest in DCIS, is also there in DCIS adjacent to IDC and IDC (Figure 5B). One of the most important findings of the present study is that *CPB1* expression pattern can differentiate an ADH lesion from a DCIS (Figure 2A), which is one of the major hurdles in BC diagnosis.

Another interesting finding from our data is the higher CPB1 expression among younger women than older women (Table 1), although this did not reach statistical significance. Of note, women with DCIS at the time of mastectomy were 46.46 ± 10.71 years old than 57.58 ± 13.74 years for women with IDC. This suggests that the DCIS might have developed around the lobular involution process and that the expression of CPB1 could be correlated with the misregulation that happens during the age-related lobular involution (ARLI) process. The ARLI involves multiple processes like apoptosis, tissue remodeling, and inflammatory processes. All of these help in the removal of non-useful epithelial cells that undergo programmed cell death and are replaced by adipose tissue and stromal cells [36]. On the other hand, all these processes also play a significant role in cancer initiation [37], and it is well documented that improper involution could lead to the onset of BC [37,38,39]. Furthermore, it has been shown that chronic inflammation can lead to improper lobular involution [40]. CPB1 is responsible for an increase in the inflammatory process by modulating the expression of proinflammatory molecules by the activation of MAPK-p38 pathway via complement system [30], which may explain its higher expression in DCIS/perimenopausal women at the time of mastectomy (high expression of CPB1 was observed among younger women, so as those diagnosed with a DCIS).

We also found that most DCIS in our cohort had microcalcifications. At the time of breast screening, the presence of microcalcification could predict the presence of an underlying DCIS [41], and microcalcification can also be associated with inflammation [42]. Our results show that the prediction of DCIS is much better with CPB1 alone than the presence of microcalcifications.

We also highlighted that loss of CPB1 in DCIS could switch the cell phenotypes to be more invasive by the loss of the tumor suppressor gene SFRP1 and *OS9* (inhibitor of HIF1α). On the other hand, loss of CPB1 leads to an increase in expression of FN1, *SPP1*, *HIF1α*, STAT3, and vimentin, indicating the reprogramming of the cells to go towards epithelial to mesenchymal transition (EMT). A study by Gauger et al. 2011 has shown that upon inhibition of SFRP1 expression, cells undergo EMT via upregulation of TGFβ signaling in BC [43]. TGFβ can induce EMT in cancer cells via upregulation of STAT3, vimentin, and FN1 [44]. On the other hand, upregulation of HIF1α also induces TGFβ mediated tumor progression [45], and both pathways can lead to BC metastasis [46].

In the present study, we identified a novel direct interactor of the CPB1 protein, TUBB3. TUBB3 is a beta-tubulin mainly expressed in nerve cells, and its dysfunction could lead to neurological disorders. Recent reports in BC suggest that the expression of TUBB3 is negatively correlated with taxane resistance [47,48,49,50,51]. Besides, TUBB3 expression is correlated with the aggressiveness of BC [52]. Our finding shows that the downregulation of CPB1 results in a decrease in the mRNA expression of *TUBB3*. Studies suggest that IDC with DCIS component shows a low recurrence rate [53], low-grade, and better survival outcome [54] and that the survival is highest when DCIS is low-grade [55]. It also has been shown that IDC with DCIS components receives chemotherapy less frequently than pure IDC [56]. However, it would be interesting to determine whether patients with IDC with DCIS components receiving taxanes show better responses than pure IDC. Nonetheless, the CPB1-TUBB3 interaction axis needs further experimental validation in BC patients.

Our study is retrospective in origin and involves a small sample size of DCIS. They both are the main limitations. As to the latter, we tried to fill the gap by providing proofs from datasets available online. However, as most datasets contain invasive tumors, further validation in a larger cohort will be needed.

## 5. Conclusions

We identified *CPB1* as a novel gene signature for DCIS. *CPB1* can help in differentiating a DCIS from an ADH lesion. More importantly, we found that loss of CPB1 in DCIS changes its phenotype towards invasiveness. Hence, *CPB1* expression, at least at the RNA level, may be useful in predicting which DCIS can progress to IDC. This information could aid clinicians and surgeons in making a priority-based decision regarding the treatment process.

Future perspectives

1. It is important to validate our finding in a larger cohort comprising ADH, ADH adjacent to DCIS, DCIS, DCIS adjacent to IDC and IDC. This will be of great importance in the clinical setting as this could truly aid clinicians in both making priority decisions and follow-up of DCIS patients.
2. It would be interesting to study whether cellular reprogramming during the involution process somehow triggers CPB1 expression leading to DCIS or tumorigenic changes due to DCIS leads to upregulation of CPB1.
3. It would be worth investigating further the relationship of CPB1 expression with microcalcifications to understand if it is merely due to the presence of microcalcifications in DCIS or if CPB1 does have any role in microcalcifications formation.

## Figures and Tables

**Table 1:** Characteristics of the study population.

Characteristics	All (*n* = 82)	Low CPB1 Expression * (*n* = 43)	High CPB1 Expression * (*n* = 39)	*p*-Value
Clinical				
Age at mastectomy (years)	55.8 ± 13.9	57.6 ± 14.0	53.9 ± 13.6	0.22
Age at menarche (years) (NA = 5)	12.8 ± 1.7	13.1 ± 1.6 (NA = 3)	12.4 ± 1.74 (NA = 2)	0.08
Menopause status (%)	51 (62.2)	32 (74.4)	19 (48.7)	0.03
Body mass index (NA = 2)	27.1 ± 5.7	28.6 ± 5.7 (NA = 2)	25.5 ± 5.30	0.01
Histopathological				
Grade ** (NA = 1)				
1	1 (1.2)	0 (0.0)	1 (2.5)	0.003
2	14 (17.1)	2 (4.76)	12 (30.8)	
3	66 (80.5)	40 (95.23)	26 (66.7)	
Tumor size (mm)	38.2 ± 27.7	44.3 ± 33.7	31.6 ± 17.1	0.03
Microcalcifications	22 (26.8)	9 (20.9)	13 (33.3)	0.30

* The two groups were divided according to the median H-score for CPB1 expression (low CPB1 expression ≤ 130, high CPB1 expression > 130), ** specific tumoral grade of the stained tissue is presented.

**Table 2:** Association between protein level of CPB1 in breast tissue and the histological types (DCIS vs. IDC).

Characteristics	Multivariate					
	Premenopausal (*n* = 31)		Postmenopausal (*n* = 51)		All (*n* = 82)	
	OR (95% CI)	*p* Value	OR (95% CI)	*p* Value	OR (95% CI)	*p* Value
CPB1 expression	1.02 (1.00–1.03)	0.022	1.03 (1.01–1.05)	0.016	1.02 (1.01–1.03)	0.00022
CPB1 expression adjusted for age at mastectomy	1.02 (1.01–1.04)	0.016	1.05 (1.02–1.14)	0.049	1.02 (1.01–1.03)	0.00056
CPB1 expression adjusted for age at mastectomy and body mass index	1.025 (1.01–1.05)	0.017	1.06 (1.02–1.15)	0.066	1.02 (1.01–1.04)	0.00069

Abbreviations: OR: odds ratio; CI: confidence interval; CPB1: carboxypeptidase B1, *p*-value < 0.05 is considered significant.

## Data Availability

Publicly available datasets were analyzed in this study. The expression data for *CPB1* in the BC dataset of the GENT2 database can be found at http://gent2.appex.kr/gent2/. This data was accessed on 5 December 2020. Survival analysis for *CPB1* and *TUBB3* were analyzed by using the dataset of KM plotter which can be found at https://kmplot.com/analysis/. The dataset was accessed for CPB1 and TUBB3 on 20 November 2020 and 6 December 2020, respectively.

## Supplementary Materials

The following are available online at https://www.mdpi.com/article/10.3390/cancers13071726/s1, Figure S1: Representative images depicting the scoring method used to quantify the IHC staining. H-score = (% of cells stained with low-intensity × 1) + (% of cells stained with medium-intensity × 2) + (% of cells stained with high-intensity × 3). DCIS (A,B), IDC (C,D). Scale bar 100 µm, Figure S2: CPB1 expression based on H-score in our cohort stratified according to grade in DCIS (A) and IDC (B), Figure S3: Association of CPB1 with microcalcification. Boxplot depicting H-score of CPB1 in patients with and without microcalcifications (A). Association of CPB1 expression in our BC cohort stratified by microcalcifications (B). ROC curve depicting the possibility of identification of DCIS based on expression of CPB1 in BC patients with microcalcifications (DCIS = 8; IDC = 3) (C) and without microcalcifications (DCIS = 4; IDC = 55) (D). Table S1: Primers used, Table S2: Clinicopathological information of patients with DCIS.
