# Supplementary material for: Is Carboxypeptidase B1 a Prognostic Marker for Ductal Carcinoma In Situ?
Source: Cancers (Basel). 2021 Apr 6;13(7):1726. doi: 10.3390/cancers13071726 (PMC8038727; doi:10.3390/cancers13071726)
Supplement: Supplementary file 1 [file cancers-13-01726-s001.pdf]

Supplementary Materials: Is Carboxypeptidase B1 a Prognostic Marker for Ductal Carcinoma In Situ?

Charu Kothari, Alisson Clemenceau, Geneviève Ouellette, Kaoutar Ennour-Idrissi, Annick Michaud, Caroline Diorio and Francine Durocher

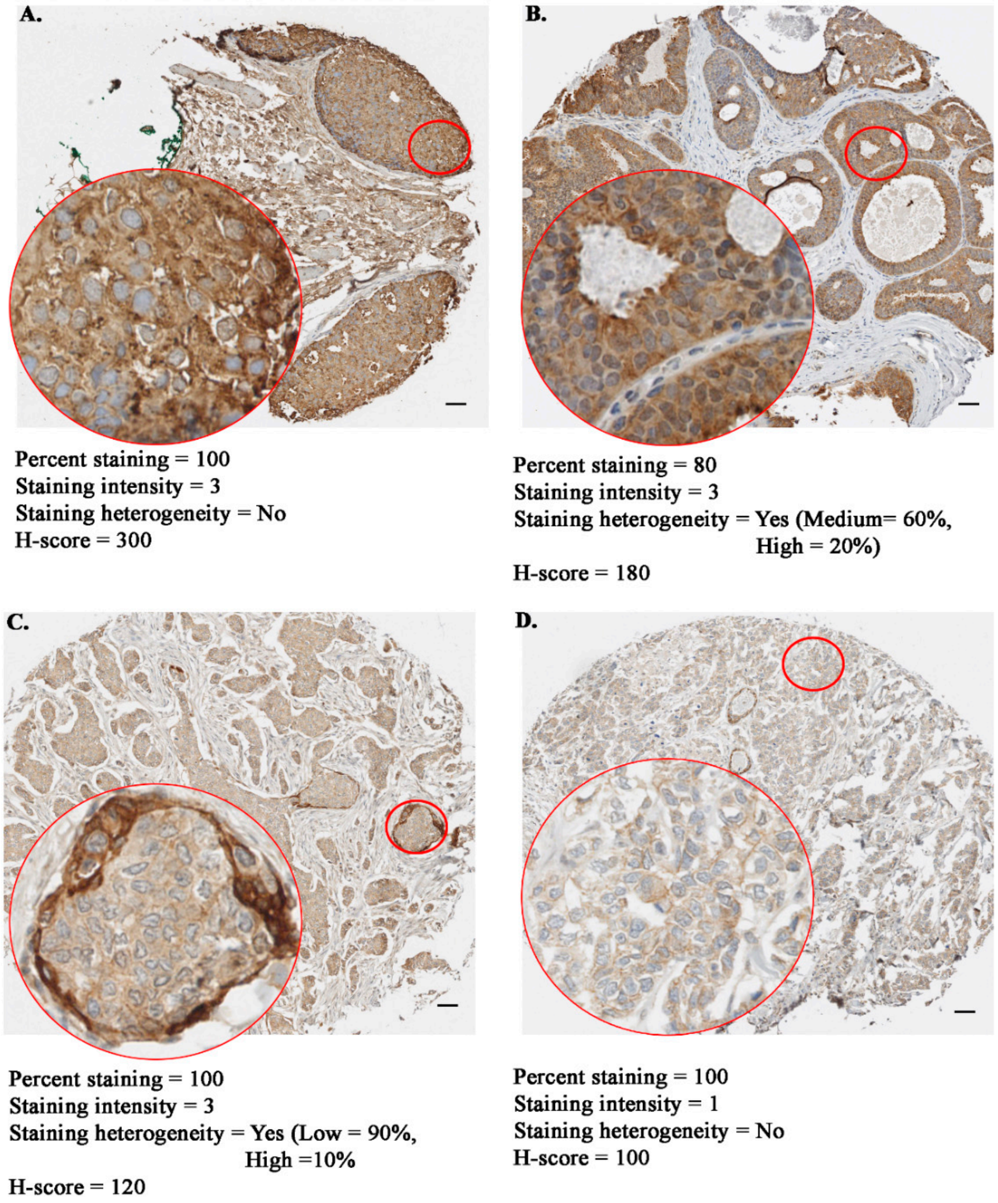

Figure S1. Representative images depicting the scoring method used for quantitation of immunohistochemistry (IHC) staining. H-score = (% of cells stained with low-intensity * 1) + (% of cells stained with medium-intensity * 2) + (% of cells stained with high-intensity * 3). carcinoma in situ (DCIS) (**A,B**), invasive ductal carcinoma (IDC) (**C,D**). Scale bar 100 μm.

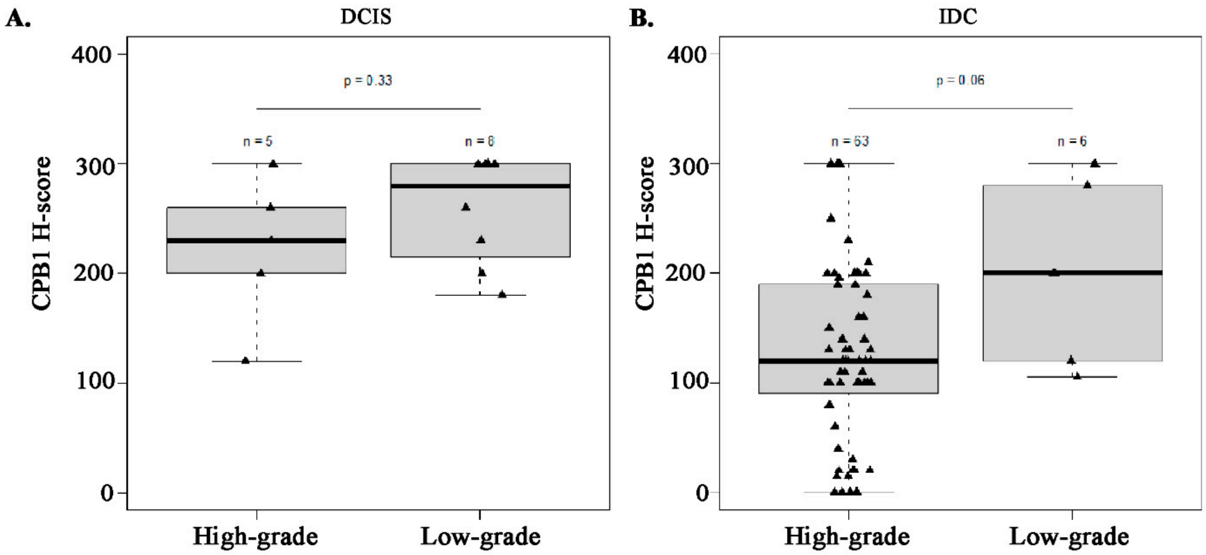

Figure S2. Carboxypeptidase 1 (CPB1) expression based on H-score in our cohort stratified according to grade in DCIS (**A**) and IDC (**B**).

A.

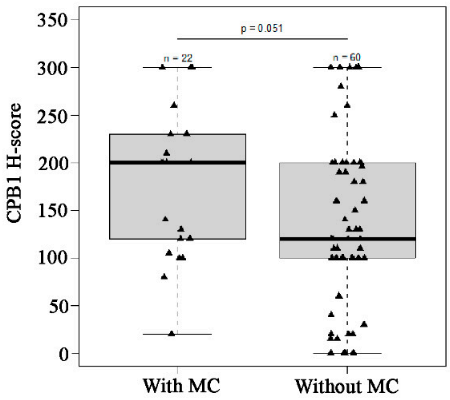

B.

Characteristics	Microcalcifications (n = 22)		No microcalcification (n = 60)		All (n = 82)	
	**OR (95% CI)**	**P value**	**OR (95% CI)**	**P value**	**OR (95% CI)**	**P value**
**CPB1 expression**	1.03 (1.01- 1.06)	0.015	1.02 (1.01- 1.05)	0.020	1.02 (1.01-1.03)	0.00022
**CPB1 expression adjusted for age at mastectomy**	1.04 (1.01- 1.09)	0.041	1.02 (1.01-1.05)	0.024	1.02 (1.01-1.03)	0.00056

C. **D.**

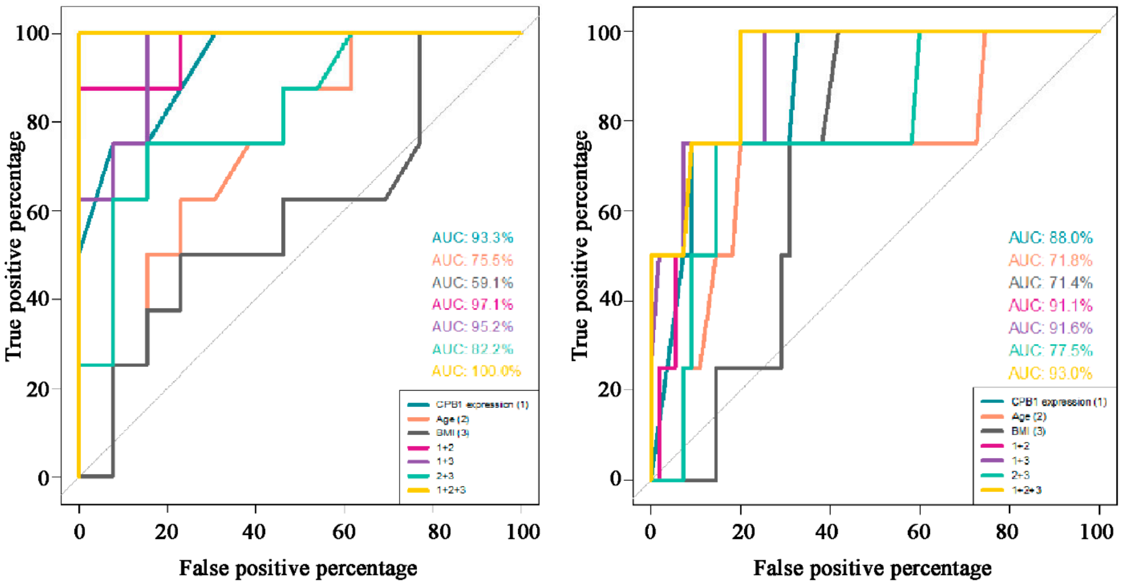

Figure S3. Association of CPB1 with microcalcification. Boxplot depicting H-score of CPB1 in patients with and without microcalcifications (**A**). Association of CPB1 expression in our BC cohort stratified by microcalcifications (**B**). Receiver operating characteristic (ROC) curve depicting the possibility of identification of DCIS based on expression of CPB1 in BC patients with microcalcifications (DCIS = 8; IDC = 3) (**C**) and without microcalcifications (DCIS = 4; IDC = 55) (**D**).

Table S1. Primers used.

Gene Symbol	Description	Size (bp)	Primer Sequence 5'→3' Forward/Reverse
**CPB1**	*Homo sapiens* carboxypeptidase B1 (CPB1)	92	TGCTGGTTGGTGTGAA ATTGGAG/ GGGCCTTGGTCTCCTTT TCAG
**SFRP1**	*Homo sapiens* secreted frizzled-related protein 1 (SFRP1)	111	TCTACCCGTGTCGCTGG CTCT/ CCTCGGGGAACTTGTC ACACTTAA
**FN1**	*Homo sapiens* fibronectin 1 (FN1), 7 transcripts	104	GCTCAAGTGGTCCTGTC GAAGTAT/ GTACTTGGAAATGTGA GATGGCTGT
**SPP1**	*Homo sapiens* secreted phosphoprotein 1 (SPP1), 5 transcripts	108	AAGAAGTTTCGCAGAC CTGACATC/ AACGGGGATGGCCTTG TATGC
**OS9**	*Homo sapiens* OS9 endoplasmic reticulum lectin (OS9), 7 transcripts	128	CCTTCCCTACAGCCTGA GGAGTA/CCAGACTTGG TCTCACTCCACAC
**HIF-1α**	*Homo sapiens* hypoxia-inducible factor 1 subunit alpha (HIF1A), 3 transcripts	144	TATGAGCCAGAAGAAC TTTTAGGCC/CCACCTC TTTTGGCAAGCATCCTG
**TUBB3**	*Homo sapiens* tubulin beta 3 class III (TUBB3), 2 transcripts	119	TCAGCGTCTACTACAA CGAGGCC/GCCTGAAG AGATGTCCAAAGGC
**ATP5O**	*Homo sapiens* ATP synthase, H+ transporting, mitochondrial F1 complex, O subunit	103	ATTGAAGGTCGCTATG CCACAG/ CCTTCAGGATTTGTGCT ACTCTCA
**HPRT1**	*Homo sapiens* hypoxanthine phosphoribosyltransferase 1	86	CGTCGTGATTAGTGAT GATGAACCA/ ACACCCTTTCCAAATC CTCAGC
**GAPDH**	*Homo sapiens* glyceraldehyde-3-phosphate dehydrogenase	93	CGGGGCTCTCCAGAAC ATCAT/ ATGCCAGTGAGCTTCC CGTTC

Table S2. Clinicopathological information of patients with DCIS.

	Age	Histological Type	Stage	Tumor Size (mm)	Grade	Lymph Nodes	BMI	Menopause Status	Microcalcifications	CPB1 H-Score	Relapse *
DCIS without IDC	34	Non-invasive	0	56	2	NA	21.5	No	In cancer cells under analysis	300	Yes
	45	Non-invasive	0	13	2	Negative	22.3	No	No microcalcification	300	No
	38	Non-invasive	0	50	2	Negative	23.5	No	No microcalcification	260	No
	42	Non-invasive	0	50	2	NA	24.2	No	No microcalcification	180	Yes (contralateral)

	43	Non-invasive	0	45	3	Negative	31.5	No	In cancer cells under analysis	260	NA
	41	Non-invasive	0	60	3	NA	NA	No	In cancer cells under analysis and in benign/normal cells	120	No
DCIS with IDC	30	Invasive	1	13	1	Negative	22.2	No	In cancer cells under analysis	200	No
	59	Invasive	1	0.5	2	Negative	20.8	Yes	In biopsy	230	Yes (DCIS, same breast)
	50	Invasive	1	6	2	Negative	19.4	No	In cancer cells under analysis	300	No
	57	Invasive	1	6	3	Negative	25.5	Yes	In benign/normal cells	200	No
	43	Invasive	2	22	2	Negative	32	No	In cancer cells under analysis	300	No
	68	Invasive	2	18	3	Positive	25.5	Yes	NA	300	No
	54	Invasive	3	40	3	Positive	30.8	Yes	In biopsy	230	Yes (IDC contralateral)

* After resection surgery.
